# Exotic *Spartina alterniflora* invasion increases CH_4_ while reduces CO_2_ emissions from mangrove wetland soils in southeastern China

**DOI:** 10.1038/s41598-018-27625-5

**Published:** 2018-06-18

**Authors:** Gui Feng Gao, Peng Fei Li, Zhi Jun Shen, Ying Ying Qin, Xi Min Zhang, Kabir Ghoto, Xue Yi Zhu, Hai Lei Zheng

**Affiliations:** 1 Key Laboratory of the Ministry of Education for Coastal and Wetland Ecosystems, College of the Environment and Ecology, Xiamen University, Xiamen, Fujian, 361102 P. R. China; 2 College of Life Sciences, Guangxi Normal University, Gulin, Guangxi, 541004 P. R. China; 3 Key Laboratory of Ecology of Rare and Endangered Species and Environmental Protection, Guangxi Normal University, Ministry of Education, Gulin, Guangxi, 541004 P. R. China; 4 Key Laboratory of Plant Physiology and Development Regulation, School of Life Science, Guizhou Normal University, Guiyang, Guizhou 550001 P. R. China

## Abstract

Mangroves are critical in global carbon budget while vulnerable to exotic plant invasion. *Spartina alterniflora*, one of typical salt marsh plant grows forcefully along the coast of China, has invaded the native mangrove habitats in Zhangjiang Estuary. However, the effects of *S*. *alterniflora* invasion on soil carbon gases (CH_4_ and CO_2_) emission from mangroves are not fully understood. Accordingly, we conducted a field experiment to investigate the soil CH_4_ and CO_2_ emission during growing seasons in 2016 and 2017 at four adjacent wetlands, namely bare mudflat (Mud), *Kandelia obovata* (KO), *Avicennia marina* (AM) and *S*. *alterniflora* (SA). Potential methane production (PMP), potential methane oxidation (PMO), functional microbial abundance and soil biogeochemical properties were measured simultaneously. Our results indicate that *S*. *alterniflora* invasion could dramatically increase soil CH_4_ emissions mainly due to the enhancement in PMP which facilitated by soil EC, MBC, TOC and *mcrA* gene abundance. Additionally, *S*. *alterniflora* invasion decreases soil CO_2_ emission. Both heterotrophic microbial respiration (*16S rRNA*) and methane oxidation (*pmoA* and *ANME-pmoA*) are responsible for CO_2_ emission reduction. Furthermore, *S*. *alterniflora* invasion greatly increases GWP by stimulating CH_4_ emissions. Thus, comparing with mangroves, invasive *S*. *alterniflora* significantly (*p* < 0.001) increases CH_4_ emission while reduces CO_2_ emission.

## Introduction

The concentrations of atmospheric carbon gases (mainly CO_2_ and CH_4_) have drastically increased since the industrial era, playing a pivotal role in global climate change^1^. Since the industrial era, the atmospheric CO_2_ concentration had increased from 280 ppm to 403.3 ppm in 2016 with an annual growth rate of 2.21 ppm^2^. In addition, by 2016, atmospheric CH_4_ concentration had reached 1853 ppb, which is 257% of the pre-industrial level^2^. Although the fossil fuel burning, cement industry and other land use changes largely enrich the atmospheric CO_2_^1^, small changes of CO_2_ emission from natural soil in long-period may also strongly alter atmospheric CO_2_ concentration^3^. For CH_4_, emission from natural ecosystems accounts for 30–40% of total CH_4_ emission^1,4^. Therefore, many studies have grown up around the theme of soil greenhouse gases emission from natural ecosystems^5–8^.

Coastal wetland ecosystems play a critical role in global carbon budget and climate change^9^. Among them, mangrove ecosystems are reported as highly productivity and most carbon rich^10,11^. As estimated, approximately 75% of total carbon of tropical mangrove ecosystems are stored in mangrove soils^11^. Therefore, many studies focused on the soil carbon gases emission from mangroves^8,12–14^. However, mangrove ecosystems are vulnerable to invasion from exotic plant species^15^. *Spartina alterniflora*, a cordgrass native to North America, has grown forcefully along the northernmost to the southernmost coast of China since it was introduced to China in 1979^16^. As a consequence, *S*. *alterniflora* invasion has become one of the most serious ecological problems, challenging the local biodiversity and ecosystem functions^17^. *S*. *alterniflora* are replacing the mangrove habitats in the recent two decades, which will be more and more serious in southeastern China in the future^16^. *S*. *alterniflora* is also one of the most productive ecosystems, as mangrove is, functioning as a large carbon sink in wetland ecosystems^11,18^.

Although many studies have been conducted to investigate the soil greenhouse gas emission from *S*. *alterniflora* salt marsh ecosystems^6,19–23^, there are very little agreements on the effects of *S*. *alterniflora* invasion on soil carbon gases emission. For instance, comparing with the native salt marsh species, *S*. *alterniflora* invasion was reported to stimulate CH_4_ emission due to its higher plant biomass^19,20,24^. In contrast, some studies showed no significant differences in greenhouse gas emissions between *S*. *alterniflora* and *Phragmites australis* stands in New England^25^. Moreover, *S*. *alterniflora* invasion was reported to increase soil CO_2_ emission while reducing CH_4_ emission in Yancheng National Nature Reserve (YNNR) in southeastern China, comparing with native *P*. *australis* marshes^21^. Up to now, inadequate attention has been paid to investigate the effects of *S*. *alterniflora* invasion on soil carbon gases emission from mangrove wetlands^8,26^. Besides, the responses of mangrove soil carbon gases emission to *S*. *alterniflora* invasion in relevant researches reached no agreement. For example, in Jiulong River Mangrove Reserve (JRMR), the soil CH_4_ emission from *S*. *alterniflora* invaded mangrove stand was much higher than that from native mangrove stands^26^. In terms of CO_2_, however, the highest emission was observed in mangrove *Sonneratia apetala* stands^26^. Even at the same locations in JRMR, much uncertainty still exists in the trend and magnitude of soil carbon gases emission after *S*. *alterniflora* invasion^8^. For instance, CH_4_ emission from *S*. *alterniflora* and mangrove *Kandelia obovata* sites are comparable, which are higher than that from bare mudflat in JRMR^8^. This inconsistency implies that the effects of *S*. *alterniflora* invasion on soil carbon cycle are complicated. As the rapid expansion of *S*. *alterniflora* in mangrove wetlands in China, there is an urgent need to reveal the soil carbon gases emission from mangroves as well as its driven factors according to *S*. *alterniflora* invasion.

Therefore, the specific objective of this study was to investigate the influences of *S*. *alterniflora* invasion on mangrove soil carbon gases emission and to explore its underlying mechanisms. It is estimated that *S*. *alterniflora* has expanded rapidly and the areal extent increased from 57.94 ha to 116.11 ha from 2003 to 2015 in Zhangjiang Mangrove Estuary^27^. The mangrove and *S*. *alterniflora* habitats in this area are currently experiencing similar tidal dynamics and soil texture, which provide our field experiment with natural advantages. Several environmental factors exhibited difference between growing season and non-growing season, including average total solar radiation (growing season: 198.24 W/m^2^; non-growing season: 144.75 W/m^2^), mean air temperature (growing season: 25.78 °C; non-growing season: 15.28 °C) and total precipitation (growing season: 513.5 mm; non-growing season: 358.9 mm). However, there is no difference in the tidal range and tidal cycle between these two seasons. Due to the higher temperature in the growing season, microbial activities and organic matter decomposition are enhanced^8,21^, which in turn affect soil carbon gases emission. Additionally, most field studies have demonstrated that a large proportion of soil carbon gases was emitted during warming seasons^6,8,26^. Similarly, our study in this particular area showed that CH_4_ and CO_2_ emission exhibited significant (*p* < 0.005) spatial difference only during the growing season (May to Oct) over the year (unpublished data). Hence, a field study was conducted during growing seasons (May, Jun and Jul) in 2016 and 2017 at Zhangjiang Mangrove Estuary to investigate soil carbon gases (CH_4_ and CO_2_) emission at four adjacent sites, namely bare mudflat (Mud), *Kandelia obovata* (KO), *Avicennia marina* (AM) and *S*. *alterniflora* (SA). Meanwhile, soil functional microbial abundance (*16S rRNA*, *pmoA*, *ANME-pmoA* and *M*. *oxyfera-pmoA*), potential methane production (PMP), potential methane oxidation (PMO), extracellular enzyme activities (invertase and β-glucosidase activity) and other biogeochemical properties (TOC, MBC and so on) were measured simultaneously.

## Results

### Spatial variations of carbon gases emission

**Figure 1:**
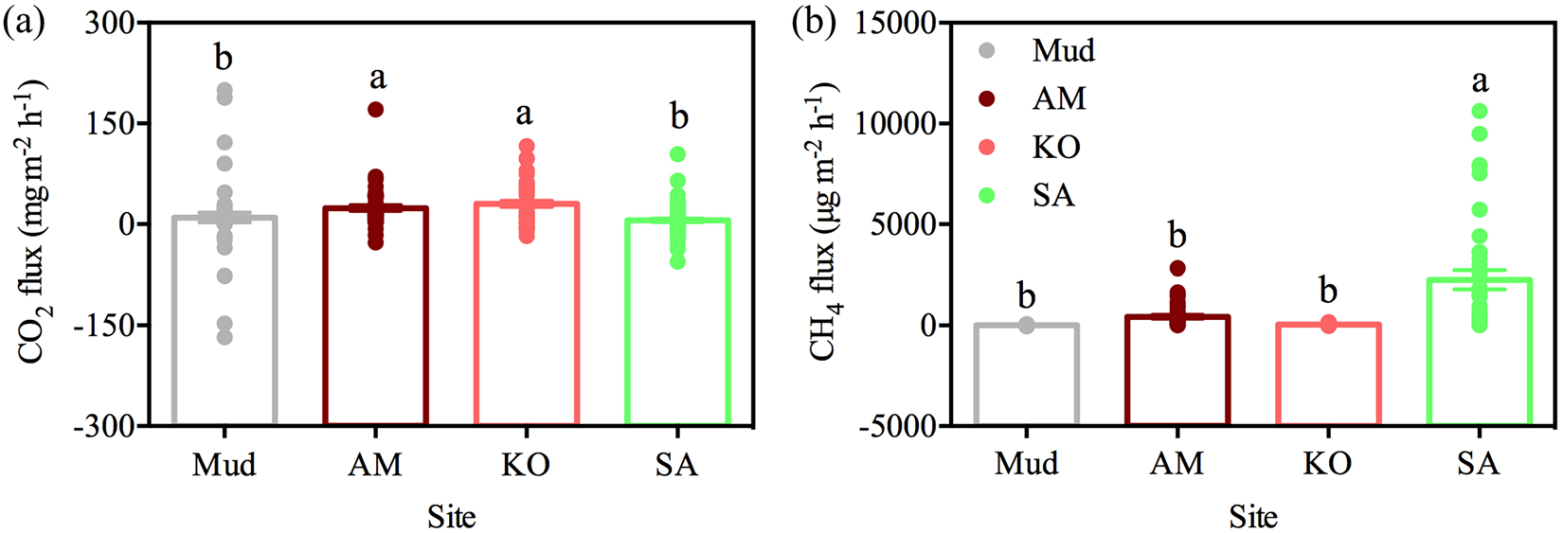
Spatial variations of CO_2_ (**a**) and CH_4_ (**b**) emission. Mud: bare mudflat; KO: *K*. *obovata*; AM: *A*. *marina*; SA: *S*. *alterniflora*. Different lowercase letters on each column indicate a significant difference (*p* < 0.05). Each point means an observation.

Figure 1 provides the results obtained from the field observations of soil carbon gases emission. On average, KO site (30.24 mg m^−2^ h^−1^) exhibited the highest CO_2_ emission, followed by AM site (23.96 mg m^−2^ h^−1^) and Mud site (9.97 mg m^−2^ h^−1^). Compared with KO, AM and Mud site, CO_2_ emission from SA site decreased by about 81.00%, 76.02% and 42.38%, respectively (Fig. 1a). For CH_4_, it showed no significant difference (*p* > 0.05) among native mangrove habitats (Mud, AM and KO). However, CH_4_ emission from SA site reached 2261.23 µg m^−2^ h^−1^, which was approximately 275, 5 and 57 times higher than those at Mud, AM and KO site, respectively (Fig. 1b).

### Differences in soil biogeochemical properties

The spatial variations of soil biogeochemical properties are shown in Table 1. In contrast to mangrove forest, *S*. *alterniflora* invasion significantly (*p* < 0.05) increased pore-water salinity, water content, soil microbial biomass carbon (MBC) and invertase activity. In addition, the lowest TC, total organic carbon (TOC), CN ratio and organic matter content were observed at Mud site, compared with AM, KO and SA site. While among those vegetation habitats (AM, KO and SA), no significant (*p* > 0.05) differences were shown. The lowest electrical conductivity (EC) was also observed at Mud site. Besides, *S*. *alterniflora* invasion decreased soil pH while mangrove species (AM and KO) showed no remarkable influence. The highest SO_4_^2−^ concentration was observed at KO and Mud site, which was significantly (*p* < 0.05) higher than that at AM and SA site. Additionally, there were no significant (*p* > 0.05) differences in soil NH_4_^+^, NO_3_^−^, NO^2−^ and β-glucosidase activity among all the mentioned sites.

**Table 1:** Soil biogeochemical properties in different sites.

Soil biogeochemical properties	Mud	KO	AM	SA
pH	7.34 ± 0.04 **ab**	7.23 ± 0.11 **ab**	7.50 ± 0.02 **a**	7.04 ± 0.11 **b**
Salinity	9.27 ± 0.02 **b**	10.63 ± 0.83 **b**	11.77 ± 0.74 **b**	14.53 ± 0.15 **a**
EC (ms/cm)	1.70 ± 0.07 **b**	1.98 ± 0.24 **ab**	2.33 ± 0.25 **ab**	2.64 ± 0.05 **a**
Water content (%)	39.39 ± 0.19 **c**	43.69 ± 1.28 **bc**	44.78 ± 0.53 **b**	50.47 ± 1.30 **a**
TC (mg/g DW)	9.31 ± 1.78 **b**	12.57 ± 0.38 **ab**	14.53 ± 0.81 **a**	12.20 ± 0.15 **ab**
TOC (mg/g DW)	8.63 ± 0.15 **b**	10.72 ± 0.12 **a**	10.94 ± 0.88 **a**	10.48 ± 0.11 **a**
CN ratio	9.03 ± 0.30 **b**	10.60 ± 0.27 **ab**	11.47 ± 0.81 **a**	9.90 ± 0.07 **ab**
Organic matter content (%)	14.81 ± 0.56 **b**	16.26 ± 0.85 **ab**	18.96 ± 0.88 **a**	18.12 ± 0.15 **a**
MBC (mg/kg DW)	59.80 ± 24.92 **c**	189.18 ± 43.63 **bc**	221.65 ± 21.30 **ab**	356.17 ± 37.63 **a**
SO_4_^2−^ concentration (mg/L)	843.18 ± 32.54 **a**	657.87 ± 24.30 **a**	423.17 ± 19.59 **b**	332.53 ± 10.89 **b**
NH_4_^+^ concentration (µg/g DW)	14.86 ± 0.24 **a**	15.12 ± 1.79 **a**	17.13 ± 1.99 **a**	16.52 ± 0.08 **a**
NO_3_^−^ concentration (µg/g DW)	1.48 ± 0.13 **a**	1.19 ± 0.02 **a**	1.08 ± 0.08 **a**	1.40 ± 0.10 **a**
NO_2_^−^ concentration (µg/g DW)	0.02 ± 0.01 **a**	0.01 ± 0.01 **a**	0.01 ± 0.01 **a**	0.01 ± 0.01 **a**
Invertase activity (mg/d/g DW)	1.12 ± 0.10 **b**	1.44 ± 0.19 **b**	2.56 ± 0.15 **b**	5.05 ± 0.88 **a**
β-glucosidase activity (mg/d/g DW)	1.53 ± 0.70 **a**	2.35 ± 0.18 **a**	2.54 ± 0.22 **a**	3.69 ± 0.75 **a**

Data are given in 0–20 cm of soil depth. Mud: bare mudflat; KO: *K*. *obovata*; AM: *A*. *marina*; SA: *S*. *alterniflora*. Different lowercase letters in each column indicate a significant difference (*p* < 0.05). TC: total carbon; TOC: total organic carbon; MBC: microbial biomass carbon; EC: electrical conductivity; DW: dry weight. Data were presented as mean ± SE.

### Shifts of functional microbial abundance based on qRT-PCR analysis

**Figure 2:**
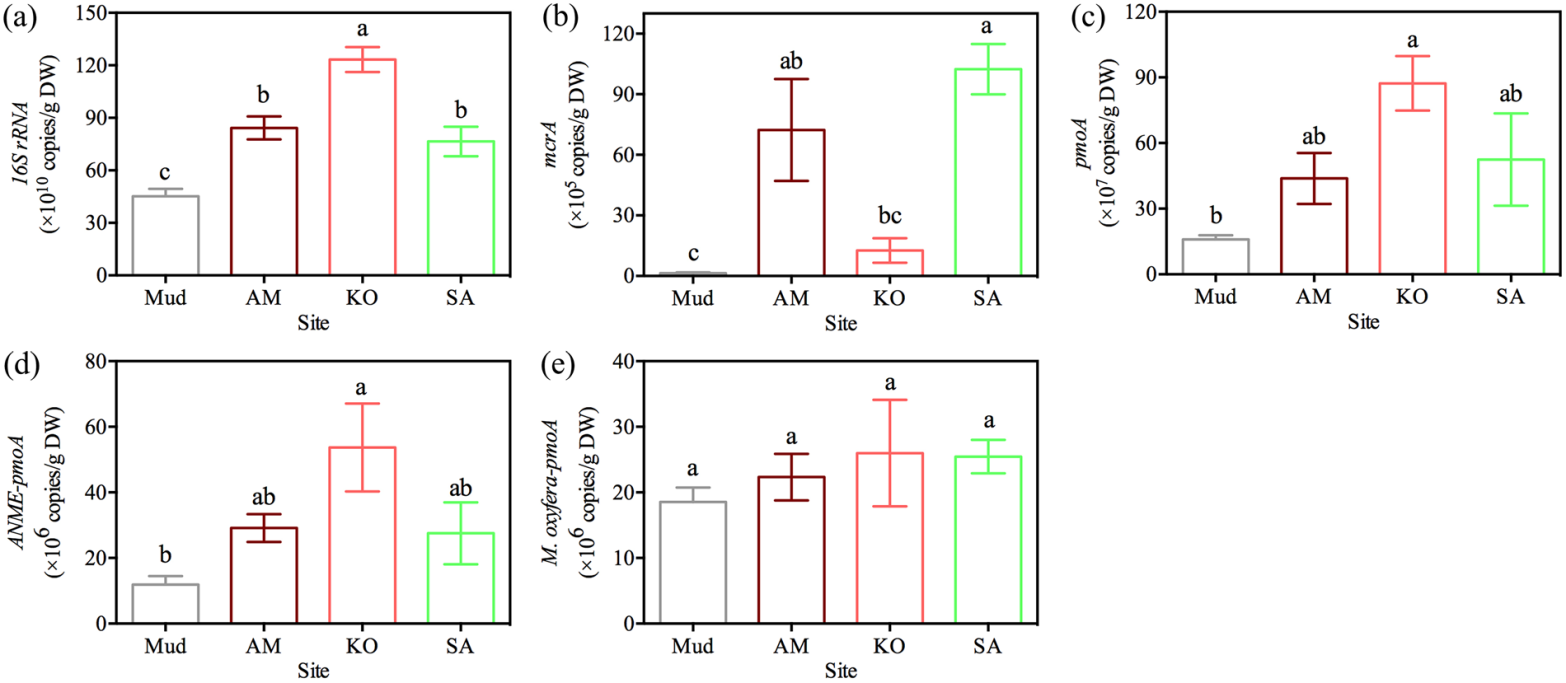
Soil functional microbial abundance *16S rRNA* (**a**); *mcrA* (**b**); *pmoA* (**c**); *ANME-pmoA* (**d)** and *M*. *oxyfera-pmoA* (**e**) in different sites. Mud: bare mudflat; KO: *K*. *obovata*; AM: *A*. *marina*; SA: *S*. *alterniflora*. DW: dry weight. Different lowercase letters in each column indicate a significant difference (*p* < 0.05). Data were presented as mean ± SE.

Soil functional microbial abundance varied from different sites, which shown in Fig. 2. Soil total microbes, which could be expressed as *16S rRNA* gene abundance, was highest at KO site and lowest at Mud site (Fig. 2a). However, the highest *mcrA* gene abundance was observed at SA site, followed by AM, KO and Mud site (Fig. 2b). About *pmoA* and *ANME-pmoA* gene abundance, the trend that be found were highest at KO site, slightly decreased at SA site and the lowest at Mud site (Fig. 2c,d). No significant (*p* = 0.68) differences were found in *M*. *oxyfera-pmoA* gene abundance among these sites (Fig. 2e).

### Changes of soil PMP and PMO

**Figure 3:**
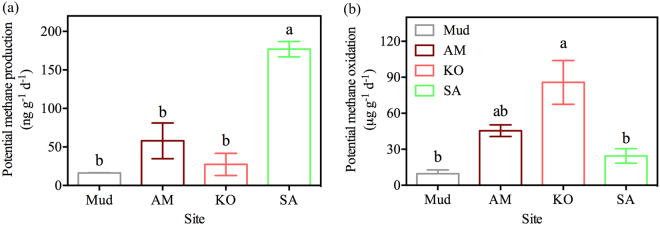
Spatial variations of potential methane production (**a**) and potential methane oxidation (**b**). Data were presented as positive values with mean ± SE. Mud: bare mudflat; KO: *K*. *obovata*; AM: *A*. *marina*; SA: *S*. *alterniflora*. Different lowercase letters on each column indicate a significant difference (*p* < 0.05).

There were significant (*p* < 0.005) differences in PMP and PMO among the sites, as shown in Fig. 3. The highest PMP was detected at SA site, reaching 176.94 ng g^−1^ d^−1^, which was about 3, 6 and 11 times higher than that at AM (58.01 ng g^−1^ d^−1^), KO (27.36 ng g^−1^ d^−1^) and Mud site (16.28 ng g^−1^ d^−1^), respectively. However, no significant (*p* > 0.10) differences in PMP were observed among AM, KO and Mud site (Fig. 3a). In contrast, PMO at SA site (24.47 ng g^−1^ d^−1^), which has no significant (*p* > 0.05) differences with Mud and AM site, was lower than that at KO (85.76 ng g^−1^ d^−1^) site (Fig. 3b).

### Estimation of annual emission of carbon gases and global warming potential (GWP)

**Figure 4:**
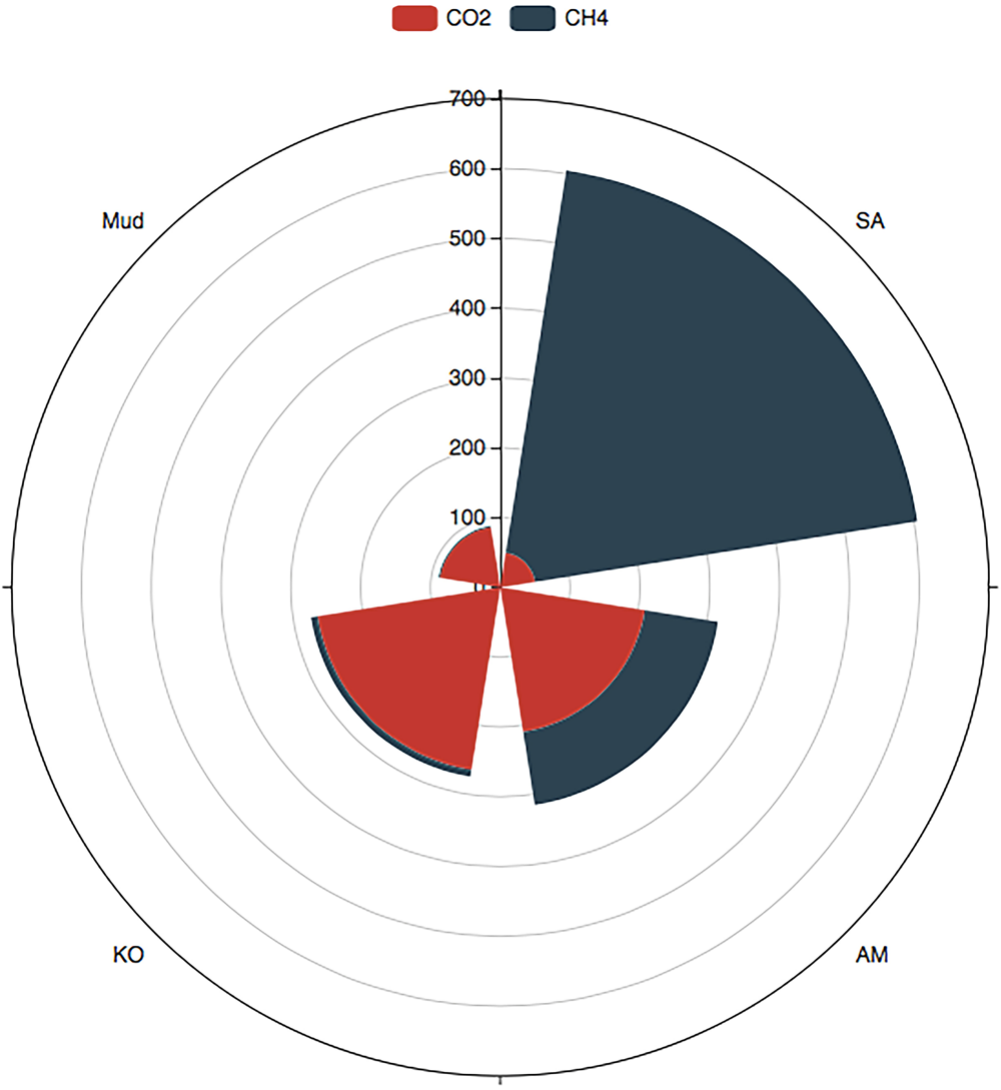
Global warming potemtial (GWP) comparision among sites. GWP was estimated as follow: GWP (g eq-CO_2_ m^−2^ year^−1^) = CO_2_ emission + CH_4_ emission * 28. Sectors in red and blue indicate CO_2_ and CH_4_ emission, respectively. Mud: bare mudflat; KO: *K*. *obovata*; AM: *A*. *marina*; SA: *S*. *alterniflora*.

In order to assess the effects of *S*. *alterniflora* invasion on GWP, we further estimated the annual emission of CO_2_ and CH_4_ based on the investigations during the growing season in the present study (Table 2, Fig. 4). We found that the level of soil CO_2_ and CH_4_ emission during the ebb tide were relatively close to the daily average emission level (Supplementary Fig. S1 on dashline), which could change over the course of the season. Therefore, the GWP was expressed as g eq-CO_2_ m^−2^ year^−1^ and calculated as following: GWP = annual CO_2_ emissions + 28 * annual CH_4_ emissions^21^. Table 2 showed that the annual CO_2_ emissions at AM, KO and Mud site were 209.80, 264.90 and 87.34 g m^−2^ year^−1^, respectively, which were higher than that at SA site (50.28 g m^−2^ year^−1^). On the contrary, in comparison with mangrove habitats, *S*. *alterniflora* invasion increased anuual CH_4_ emission remarkably, estimated as 19808.37 mg m^−2^ year^−1^. As calculated and shown in Fig. 4, the GWP was highest at SA site (604.91 g eq-CO_2_ m^−2^ year^−1^), which was approximately 1.9, 2.2 and 6.8 times higher than that from AM, KO and Mud, respectively. CH_4_ emission only accounts for 2.3–33.5% of total GWP at native mangrove habitats, while this proportion reached 91.7% at SA site.

**Table 2:** Estimation of annual emission of CO_2_ (g m^−2^ year^−1^) and CH_4_ (mg m^−2^ year^−1^) based on carbon gases emission measurements in the present study during growing season in 2016 and 2017.

Annual emission	Mud	AM	KO	SA
CO_2_ (g m^−2^ year^−1^)	87.34	209.80	264.90	50.28
CH_4_ (mg m^−2^ year^−1^)	71.92	3780.38	346.11	19808.37

Mud: bare mudflat; KO: *K*. *obovata*; AM: *A*. *marina*; SA: *S*. *alterniflora*.

## Discussion

The current study found that *S*. *alterniflora* invasion drastically increased CH_4_ emission by 5–275 folds, which was significantly (*p* < 0.0001) higher than that from native mangrove habitats (Fig. 1b). Our results were well in agreement with the study conducted in JRMR which revealed that *S*. *alterniflora* site has the highest soil CH_4_ emission, comparing with mangrove *K*. *obovata* and *S*. *apetala* site^26^. However, Wang and co-authors found no significant differences in soil CH_4_ emission between *S*. *alterniflora* and *K*. *obovata* site in JRMR^8^. In salt marshes ecosystem of southeastern China, *S*. *alterniflora* invasion was reported to increase soil CH_4_ emission, comparing with *P*. *australis* in YNNR^6^ and *Cyperus malaccensis* in Shanyutan wetland^20^ as well as *Scirpus mariqueter* in Yangtze River estuary^19^. Previous studies concluded that *S*. *alterniflora* stimulated soil CH_4_ emission mainly due to its higher plant biomass than that of native salt marsh plant^20,24^. Nevertheless, soil CH_4_ emission from *S*. *alterniflora* site was slightly lower than *P*. *australis* site in YNNR^21^ or comparable with *P*. *australis* site in New England^25^. Many abiotic factors, such as salinity which influencing the activities of methanogens^28^, inorganic nitrogen (NH_4_^+^ and NO_3_^−^) which impacting CH_4_ oxidation^29,30^, can influence soil CH_4_ emission. Other than that, soil functional microbes play an important role in regulating soil CH_4_ dynamic^31^. In JRMR, *S*. *alterniflora* invasion was reported to increase soil bacterial richness and change microbial community structure^32^. Therefore, we inferred that the distinct effects of *S*. *alterniflora* invasion on soil CH_4_ emission may be mainly due to the highly discrepancy in soil functional microbial abundance (Fig. 2).

**Figure 5:**
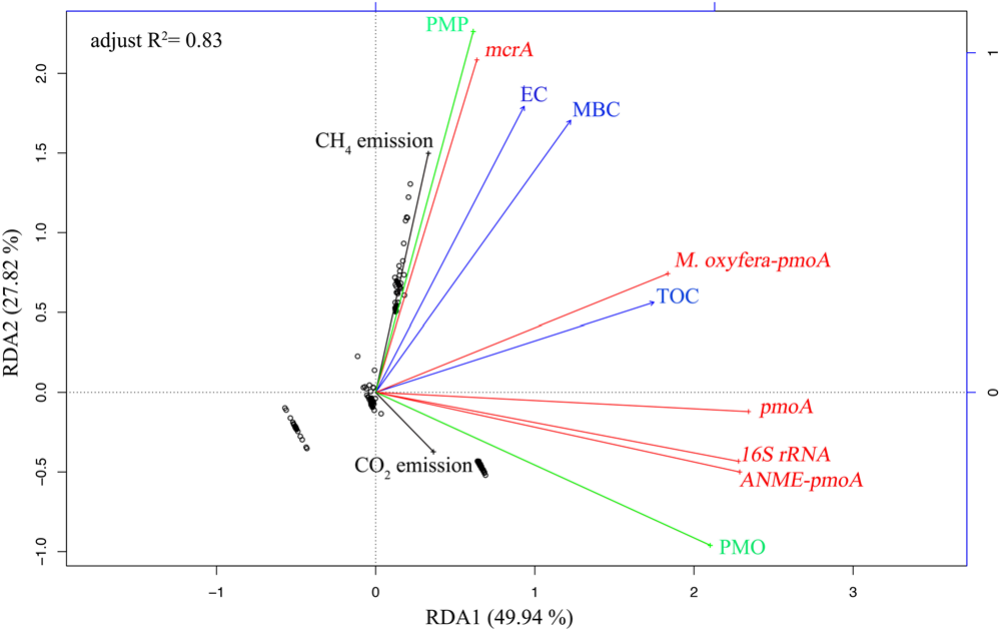
Permutation (number: 9999) test for redundancy analysis (RDA) under reduced model of carbon gases emission, PMP, PMO and functional microbial abundance with enviromental biogeochemical properties. TOC: total organic carbon; EC: electrical conductivity; MBC: microbial biomass carbon; PMO: potential methane oxidation; PMP: potential methane production. Lines in black indicate carbon gases emission. PMO and PMP were presented as green color. Functional microbial abundance was marked as italic and red lines. Soil biogeochemical properties were showed in blue lines with arrows.

Based on redundancy analysis (RDA), CH_4_ emission was strongly positively correlated with PMP (Fig. 5). This relationship between CH_4_ emission and PMP was also found in the rice paddy field at Shizukuishi, Japan^33^. Nevertheless, the PMP showed no spatial differences and was not correlated with soil CH_4_ flux in Great Xing’an Mountains^34^. This inconsistency may be due to the subtle environmental differences as well as the complex processes of CH_4_ emission. Our results showed that *S*. *alterniflora* invasion enhanced PMP by 3–11 folds (Fig. 3a). In salt marsh ecosystem, *S*. *alterniflora* invasion was reported to increase PMP^20,35^. Firstly, the PMP was positively correlated with soil TOC (Fig. 5). Previous studies also observed a significant correlation between PMP and soil organic carbon in YNNR^35^. In our study, TOC were significantly (*p* < 0.05) higher at vegetation site (AM, KO and SA) than that at Mud site (Table 1). Liu *et al*. (2007) reported that TOC in *S*. *alterniflora* salt marshes was significantly higher than that in mudflats in Jiangsu^36^. Other relative studies have demonstrated that *S*. *alterniflora* could enhance TOC storage comparing with native salt marshes^37,38^ and mangrove *K*. *obovata*^26^. However, TOC showed no significant (*p* > 0.80) spatial differences among AM, KO and SA site (Table 1). This may be explained by the facts that the carbon sequestration rates were comparable between mangroves and *S*. *alterniflora* salt marshes^39^. Secondly, the PMP was enhanced as the MBC increased after *S*. *alterniflora* invasion (Fig. 5). It was reported that *S*. *alterniflora* invasion significantly increased MBC by increasing soil available substrates^40^. MBC is an important factor in the regulation of soil carbon cycle^41^. Thirdly, EC, as an important indicator of total soluble salt, was positively correlated with PMP (Fig. 5). Previous studies in *S*. *alterniflora* salt marshes in eastern China demonstrated that EC was significantly positively correlated with MBC and TOC^40^. In our study, we found higher EC in *S*. *alterniflora* sites than that in mangrove sites (Table 1), indicating that *S*. *alterniflora* invasion could supply methanogens with more available substrates, such as ‘non-competitive’ substrate trimethylamine^6^. Previous study found that CH_4_ production was primarily associated with trimethylamine in *S*. *alterniflora* site^35^. Fourthly, PMP was also influenced by methanogens (*mcrA*) abundance. The increase in *mcrA* abundance will lead to an enhancement of PMP (Fig. 5). In our study, *S*. *alterniflora* invasion significantly (*p* < 0.005) increased the *mcrA* abundance (Fig. 2b), which matched the conclusions in earlier studies in coastal salt marsh in YNNR^35^ and in Dongtan^42^. It was reported that methanogen community was regulated by trimethylamine which produced by *S*. *alterniflora*^35^. Besides, soil TOC may facilitate the growth of methanogens population^43^.

In our study, comparing with native mangrove KO and AM habitats, *S*. *alterniflora* invasion decreased PMO (Fig. 3b), which was negatively correlated with soil CH_4_ emission (Fig. 5). Generally, the increased PMO activity will directly reduce the CH_4_ emission^44,45^. On one hand, high PMO occurred after *S*. *alterniflora* invasion due to its highly development of aerenchyma tissue which conducted more O_2_ into the rhizosphere, inducing more CH_4_ oxidation^20^, on the other hand, the aerenchyma structure of *S*. *alterniflora* can facilitate the soil CH_4_ transportation to atmosphere^46^. In our study, the increases of CH_4_ produced by *S*. *alterniflora* may emit through aerenchyma, finally resulting in high soil surface emission in SA site. *S*. *alterniflora* invasion increased soil water content in salt marsh in YNNR^47^, similar results were also obtained in our study. The high water content may cause an anoxic condition and reduce aerobic CH_4_ oxidation. In addition, the SO_4_^2−^ concentration showed higher at Mud and KO sites than that at AM and SA sites (Table 1). SO_4_^2−^, as the electron acceptor, is associated with sulfate-dependent anaerobic CH_4_ oxidation^48^. Furthermore, *S*. *alterniflora* invasion decreased both *pmoA* and *ANME-pmoA* gene abundance, indicating that less CH_4_ oxidation occurred after *S*. *alterniflora* invasion (Fig. 3b). Taken together, comparing with native mangrove species, *S*. *alterniflora* invasion significantly (*p* < 0.05) increased soil EC, MBC, TOC and *mcrA* gene abundance which collectively enhanced PMP. Correspondingly, *S*. *alterniflora* invasion decreased *ANME-pmoA* and *pmoA* gene abundance, which decreased PMO.

In our study, *S*. *alterniflora* invasion was found to decrease soil CO_2_ emission (Fig. 1a, Table 2). The CO_2_ emission at SA sites was significantly (*p* < 0.01) lower than mangrove KO and AM habitats while was comparable (*p* = 0.91) to Mud site (Fig. 1a). However, our study did not agree with previous studies which suggested that *S*. *alterniflora* had higher CO_2_ emission than mangrove *K*. *obovata* site in JRMR^8,26^. Besides, there were no changes in CO_2_ emission after *S*. *alterniflora* invasion into Yangtze River estuarine wetland^49^. It is widely known that CO_2_ emission is mainly from root respiration and heterotrophic microbial respiration in the soil. Relatively, heterotrophic microbial respiration was found to be the main source of soil CO_2_ emission^50^. The effects of *S*. *alterniflora* on soil CO_2_ emission in our study can be explained as following. Firstly, soil CO_2_ emission was positively correlated with *16S rRNA* gene abundance and soil TOC in Tibetan alpine meadow^51^, similar to our results (Fig. 5). Soil CO_2_ emission derives from the decomposition of organic substances^3^. Therefore, soil CO_2_ emission is at least partially influenced by soil TOC content and its mineralization^52^. Chen and co-authors found that, in the mangrove ecosystem, soil CO_2_ emission was positively correlated with TOC^12,26^, similar as the results in our study (Fig. 5). However, other studies showed that soil CO_2_ emission was negatively correlated with TOC in North Sulawesi mangrove swamps^13^. In conclusion, on one hand, as highly productive and biomass-rich ecosystem, mangrove species may provide more TOC for soil microbes (Table 1). On the other hand, high *16S rRNA* gene abundance was found at mangrove stands (Fig. 2a). Thus, high abundance of the *16S rRNA* gene and high TOC may collectively support the high CO_2_ emission in mangrove habitats (Fig. 1a). Secondly, a large proportion of produced CH_4_ was oxidized to CO_2_, especially in marine soil^31,53^. A strongly positive relationship between CO_2_ emission and PMO showed an important role of CH_4_ oxidation in CO_2_ emission (Fig. 5). Tong *et al*. (2012) found that the CH_4_ oxidation rate was higher at *S*. *alterniflora* site than that at *C*. *malaccensis* site in Shanyutan wetland^20^, while we characterized that the PMO decreased after *S*. *alterniflora* invasion comparing with mangrove habitats (Fig. 3b). *S*. *alterniflora* absorb SO_4_^2−^ for its growth, resulting in low concentration of SO_4_^2−^ (Table 1). High SO_4_^2−^ at mangrove stands may cause more sulfate-dependent anaerobic CH_4_ oxidation^48^. In addition, *pmoA* and *ANME-pmoA* gene abundance was also higher at mangrove KO stands, indicating that mangrove habitats have higher CH_4_ oxidation, especially the CH_4_ oxidation associated with SO_4_^2−^ reduction (Fig. 2c,d). Overall, *S*. *alterniflora* invasion decreased CO_2_ emission compared with native mangrove habitats. The combinations of heterotrophic microbial respiration (*16S rRNA*) and anaerobic methane oxidation (*pmoA* and *ANME-pmoA*) were the main factors in regulating CO_2_ reduction.

We suggest that *S*. *alterniflora* invasion is a double-edged sword. On one hand, *S*. *alterniflora* have expanded vastly from 57.94 ha to 116.11 ha during 2003–2015 in Zhangjiang mangrove estuary^27^. Due to its high productivity, this large-scale of *S*. *alterniflora* plant community shift is expected to allocate more atmospheric CO_2_^23^. Comparing with mangrove ecosystem, *S*. *alterniflora* invasion enhanced soil carbon stock (TC, TOC, CN ratio, organic matter content and MBC) (Table 1). On the other hand, *S*. *alterniflora* invasion decreased soil CO_2_ emission while significantly (*p* < 0.0001) increased soil CH_4_ emission (Fig. 1, Table 2). The estimation of GWP was drastically increased by *S*. *alterniflora* invasion (Fig. 4). Xu *et al*. (2014) reported that in YNNR, *S*. *alterniflora* site has the highest GWP^21^. In our study, the estimation of GWP may be over-estimated since we calculated only based on the emission data during the growing season. Nevertheless, our results indicated the important effects of *S*. *alterniflora* invasion on soil carbon dynamics as well as the regional GWP. Further studies aiming at the impacts of *S*. *alterniflora* invasion, particularly by root exudations, on mangrove soil microbial community structure and then C dynamics should be focused.

## Materials and Methods

### Study site

**Figure 6:**
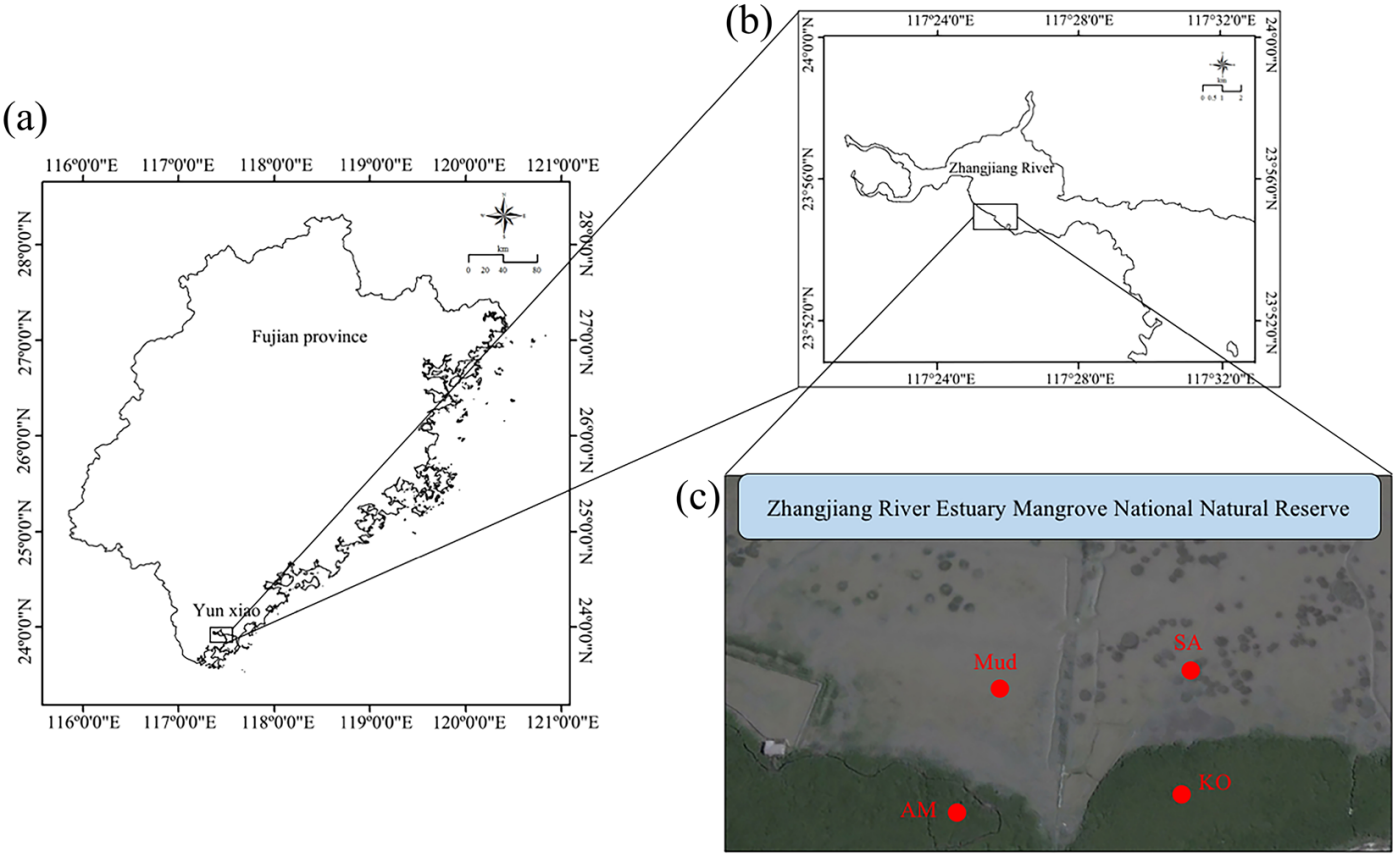
Map description of the study site. Map of Fujian Province and the location of Yunxiao County which marked as black square (**a**); the selected study area (marked as black square) in Zhangjiang River Estuary Mangrove National Natural Reserve (**b**); red points indicate the sample site location in Zhangjiang River Estuary Mangrove National Natural Reserve. Mud: bare mudflat; KO: *K*. *obovata*; AM: *A*. *marina*; SA: *S*. *alterniflora*. Base maps (**a**,**b**), scale bars and the longitude and latitude were added using ArcGIS for desktop version 10.3 (http://www.esri.com). Image (**c**) was generated from Google Earth (image source: Google, Landsat/Copernicus, copyright DigitalGlobe). Images were arranged using Adobe Photoshop CC 2017 version 18.0.1 (http://www.adobe.com).

Our study was conducted at Zhangjiang River Estuary Mangrove National Natural Reserve in Yunxiao County, Fujian Province, China (117°24′–117°30′E, 23°53′–23°56′N) (Fig. 6). This region is subjected to subtropical marine monsoon with an annual average temperature of 21.2 °C and annual average precipitation of 1714 mm. This area experiences typical semi-diurnal tide with a mean tidal range of 2.32 m^54^. In addition, this area situates at the outlet of Zhangjiang River with an average river discharge of about 1,011,220,000 m^3^ per year^27^. The total area of this natural reserve is 2360 ha, which consists of 116.11 ha of the invasive plant species *S*. *alterniflora* (SA)^27^. Besides, native mangrove species *K*. *obovata* (KO) and *A*. *marina* (AM) are widely distributed in this area^55^. Therefore, four experimental sites with three replicates each were randomly established, including bare mudflat (Mud), KO, AM and SA, representing different vegetation types. The vegetation at each site was monoculture and was at least 20 m away from each other to eliminate the edge effects. All sites were experiencing similar elevation and exposure time to reduce the tidal influences during the field experiment period.

### Carbon gases sampling and quantification

In order to obtain daily average soil CO_2_ and CH_4_ emission, a pilot experiment was conducted in KO and AM sites during two complete tidal cycles in Oct 2014. Three replicates for each site were applied in carbon gases emission measurements using static closed chamber coupled with gas chromatography^12^. PVC chambers with the volume of 2 L and the diameter of 20 cm (total area coverage of 0.031 m^2^) were applied in sampling, similar to Chen *et al*.^12^. Before sampling, a foam board was installed at each site to diminish the anthropogenic impacts. Gas samplings were conducted at neap tide during 12:00–14:00 local time as predicted by China Shipping Service (CNSS). During each sampling period, above-ground mangroves or *S*. *alterniflora* vegetation were not covered in chambers. The sampling procedures in the present study were similar to Chen *et al*. (2010) with small modification^56^. In brief, the open end of the chamber was lightly inserted 3 cm into the soil, then were removed to make a groove, which are used to sealed the chamber in case of the gas leakage. After soil stabilization for 20 mins, the chamber was recovered again over the soil. At 0, 5, 10 and 15 min after closure, headspace air was mixed carefully and 10 ml mixed air was collected by passing a 100 ml gas-tight glass syringe through the sampling outlet. Gas samples were then injected into clean fluorinated ethylene propylene (FEP) Teflon air bags immediately. Temperature inside the chamber was measured simultaneously. In mangrove and *S*. *alterniflora* ecosystems, soil GHGs emissions were usually calculated and well represented by using 3 time points^6,12,24,57^. Although our pilot experiment showed that the obtained gas fluxes by using these approaches were comparable with those data by Jin *et al*.^58^, these operations need to be proceed very carefully, or it may cause bias in gases concentrations inside chamber.

Gas samples were analyzed within 24 hours using gas chromatograph (Agilent 7890B, Santa Clara, CA, USA) equipped with a flame ionization detector (FID) with pure nitrogen as the carrier gas. The gas concentrations were determined by comparing the sample peak areas against the standard curves (National Research Center for Certified Reference Materials, Beijing, China). To assure data quality, gradient standard gases were inserted into the gas chromatograph system every hour. All gas concentrations showed confident linear relationship with closure time. Therefore, gas emission rates were calculated using a linear least squares fit^6^. Data were accepted if the slope of the linear fitting had R^2^ > 0.90. Based on our observations, the data collected from 2016 and 2017 were comparable for each site. Hence, the obtained data were combined for analysis. Besides, we defined the net soil CO_2_ flux as soil CO_2_ emission, which including both plant dark respiration and soil basal respiration.

### Soil biogeochemical properties measurements

For each site, three independent soil cores (0–20 cm) were randomly collected using a 6.0 cm diameters PVC tube in Jul 2016. Intact soil cores were then sealed in black plastic bags immediately and stored with ice during the transportation. Before analysis, plant roots and other debris were removed and the remaining soil was completely homogenized. Pore-water salinity was determined after centrifugation of soil using a MASTER-S/MillM salinity refractometer (ATAGO, Japan). Pore-water SO_4_^2−^ concentration was measured using a turbidimetric method according to the methods proposed by Cáceres and colleagues^59^. Soil inorganic N (NH_4_^+^, NO_3_^−^ and NO^2−^) concentration were analyzed using AA3 Auto Analyzer 3 (Seal, Germany) after extracted with 2 mol/L KCl as described by Chen *et al*.^12^. The chloroform fumigation-extraction method was employed to measure soil MBC^47^. Traditionally, soil water content was determined by oven-drying of 50 g fresh soil at 30 °C to a constant weight^60^. After that, dried soil was ground and sieved through a 2 mm sieves. Soil pH was measured at a *w* (soil): *v* (water) of 1: 5 using an Orion 3 star digital portable pH meter with a precision level of ±0.01 (Thermo, USA). Organic matter content was measured based on the loss on ignition at 550 °C for 6 hours after 105 °C oven-dried and calculated as OM = ((DW_105 °C_ − DW_550 °C_)/DW_105 °C_) * 100, where DW denotes dry weight^61^. Before TOC measurements, soil samples were decarbonized with 1 mol/L HCl^62^. Total carbon (TC), TOC and CN ratio were then determined using a Vario EL III Elemental Analyzer with a precision level of ±0.01 for C and N determination (Elementar, Hanau, Germany). In addition, soil invertase activity and β-glucosidase activities were measured according to Shao *et al*.^41^. Soil EC was measured at 25 °C using a conductivity meter (Leici DDS-307, Shanghai, China)^40^.

### Soil DNA extraction and quantitative real-time PCR assay

Soil DNA was extracted with FastDNA Spin Kit for Soil (MP Biomedical, Carlsbad, USA) and stored at −20 °C for further experiments. DNA quality and quantity were examined with Nanodrop ND-1000 spectrophotometer (PeqLab, Germany) and agarose gel electrophoresis. A quantitative real-time polymerase chain reaction (qRT-PCR) method was established and applied to determine soil functional microbial abundance based on *16S rRNA*^63^, *mcrA*^64^, *pmoA*^44^, *ANME-pmoA*^65^ and *M*. *oxyfera-pmoA*^66^ (Supplementary Table S1). All qRT-PCR reactions were performed on Bio-Rad CFX96 machine in three replicates for each sample. 20 µL of each reaction mixture contained 10 µL TransStart Tip Green qPCR SuperMix (TransGen Biotech, Beijing), 1 µL DNA, 0.4 µL of each primer and 8.2 µL of nuclease free H_2_O. PCR products generated using M13 primers from plasmids were used for standard curves. PCR programs: initialization at 95 °C for 5 min, 40 cycles of denaturation at 95 °C for 30 s, annealing for 30 s and final extension at 72 °C for 30 s.

### Potential methane production (PMP) and potential methane oxidation (PMO) measurements

PMP and PMO were determined according to Robroek *et al*.^67^. In brief, for PMP measurements, 10 g fresh soils were incubated in 120 mL glass vials with a serum cap containing 10 mL sterilized demineralized water. The vials were sealed and the slurry was mixed evenly. All vials were then removed to an incubator under dark after purging with pure N_2_ for 20 minutes. For PMO measurements, 10 g fresh soils were incubated in 120 mL glass vials containing 10 mL sterilized demineralized water. Headspace air was replaced with 2 mL CH_4_ (10000 ppmv) to ensure the sufficient methane throughout the experiment. The glass vials were then incubated on a shaker with 150 rpm at 20 °C. Headspace CH_4_ were sampled every 24 hours for 10 days. For CH_4_ quantification, headspace gas samples were injected into gas chromatograph (Agilent 7890B, Santa Clara, CA, USA) as described previously. PMP and PMO were calculated from the linear changes of CH_4_ concentrations and presented as positive values.

### Statistics analysis

Figures were performed in GraphPad Prism (Version 6.0c, GraphPad Software, California, USA) or ECharts (http://echarts.baidu.com). Data normality and homogeneity of variance were checked using Shapiro-Wilk normality test and Bartlett test, respectively (R Studio, 1.0.44). The ordinary one-way ANOVA analysis followed by Tukey’s multiple comparisons test was performed to compare the spatial difference in soil carbon gases emission, functional microbial abundance, biogeochemical properties, PMP and PMO when data passed normality and homogeneity of variance tests (SPSS, 22.0). Otherwise, non-parametric (Kruskal-Wallis) analysis followed by Dunn’s multiple comparisons test were used (SPSS, 22.0). In addition, we used a permutation test (permutations number: 9999) for redundancy analysis (RDA) under reduced model to test the primarily environmental factors that drive the variations of soil carbon gases emission, functional microbial abundance, PMP and PMO (R Studio, Version 1.0.44). Significance was accepted if *p* < 0.05 for all analysis.

### Data availability

Additional data can be found in the supplementary material. All data are available upon request by email to the corresponding author.

## Electronic supplementary material

Supplemental information
