# Supplementary material for: Exotic Spartina alterniflora invasion increases CH4 while reduces CO2 emissions from mangrove wetland soils in southeastern China
Source: Sci Rep. 2018 Jun 18;8:9243. doi: 10.1038/s41598-018-27625-5 (PMC6006287; doi:10.1038/s41598-018-27625-5)
Supplement: Supplementary file 1 — Supplemental information [file 41598_2018_27625_MOESM1_ESM.pdf]

Title: Exotic *Spartina alterniflora* invasion increases CH_4 while reduces CO_2 emissions from mangrove wetland soils in southeastern China

Authors: Gui Feng Gao[a], Peng Fei Li[a], Zhi Jun Shen[a], Ying Ying Qin[a,b,c], Xi Min Zhang[a,d], Kabir Ghoto[a], Xue Yi Zhu[a] and Hai Lei Zheng[a,*]

[a] Key Laboratory of the Ministry of Education for Coastal and Wetland Ecosystems, College of the Environment and Ecology, Xiamen University, Xiamen, Fujian 361102, PR China

[b] College of Life Sciences, Guangxi Normal University, Gulin, Guangxi 541004, PR China

[c] Key Laboratory of Ecology of Rare and Endangered Species and Environmental Protection, Guangxi Normal University, Ministry of Education, Gulin, Guangxi 541004, PR China

[d] Key Laboratory of Plant Physiology and Development Regulation, School of Life Science, Guizhou Normal University, Guiyang, Guizhou 550001, PR China

***Corresponding author:**

Hai-Lei Zheng

Tel: +86 592-218-1005

Fax: +86 592-218-5889

E-mail: zhenghl@xmu.edu.cn

Table S1 Primers sequences of *16S rRNA*, *mcrA*, *pmoA*, *ANME-pmoA* and *M.oxyfera-pmoA* gene used in this study.

Genes	Primers sequences (5'-3')	TM (°C)	Length (bp)	Reference
16S rRNA	16s rRNA-F: ACTCCTACGGGAGGCAGCAG<br>16s rRNA-R: ATTACCGCGGCTGCTGG	55	195	63
mcrA	mcrA F: GGTGGTGTMGGATTCACACARTAYGCWACAGC<br>mcrA R: TTCATTGCRTAGTTWGGRTAGTT	51	488	64
pmoA	A189 F: GGNGACTGGGACTTCTGG<br>Mb661 R: CCGGMGCAACGTCYTTACC	65	508	44
ANME-pmoA	337f: AGGTCCTACGGGACGCAT<br>724r: GGTCAGACGCCTTCGCT	58	357	65
M. oxyfera-pmoA	qP1F: GGGCTTGACATCCCACGAACCTG<br>qP1R: CGCCTTCCTCCAGCTTGACGC	65	289	66

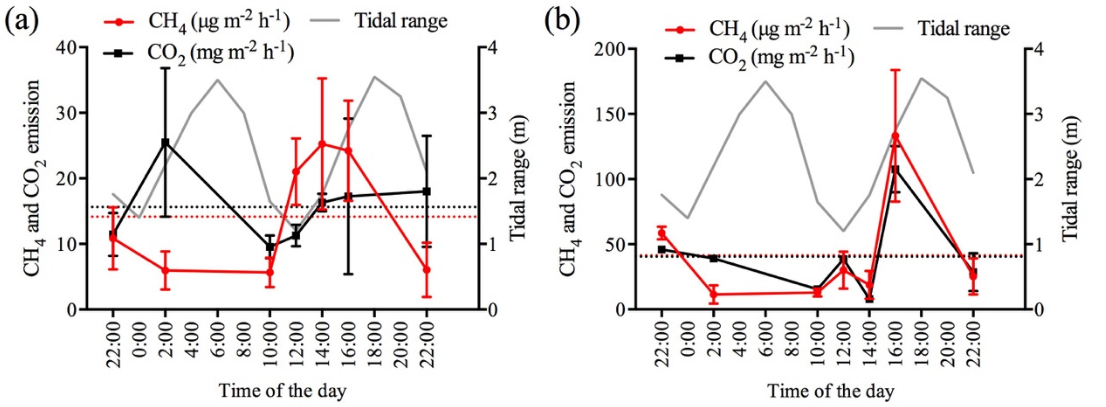

Fig. S1 Daily variations of soil CH_4 and CO_2 emissions from *Kandelia obovata* (a) and *Avicennia marina* (b) site in 2-hour intervals. Data were presented as mean ± SE. The horizon dash lines in red and black indicate the mean values of soil CH_4 and CO_2 emissions, respectively.
